# Aberrant DNA methylation associated with silencing BNIP3 gene expression in haematopoietic tumours

**DOI:** 10.1038/sj.bjc.6602422

**Published:** 2005-03-08

**Authors:** M Murai, M Toyota, A Satoh, H Suzuki, K Akino, H Mita, Y Sasaki, T Ishida, L Shen, G Garcia-Manero, J-P J Issa, Y Hinoda, T Tokino, K Imai

**Affiliations:** 1 First Department of Internal Medicine, Sapporo Medical University, Sapporo 060-8556, Japan; 2 Department of Molecular Biology, Cancer Research Institute, Sapporo Medical University, Sapporo 060-8556, Japan; 3 PRESTO, JST, Kawaguchi, 332-0012, Japan; 4 Department of Leukemia, MD Anderson Cancer Center, Houston, TX 77030, USA; 5 Division of Clinical Laboratory, Yamaguchi University School of Medicine, Ube 755-8505, Japan

**Keywords:** DNA methylation, epigenetics, chromatin

## Abstract

Hypoxia is a key factor contributing to the progression of human neoplasias and to the development of resistance to chemotherapy. BNIP3 is a proapoptotic member of the Bcl-2 protein family involved in hypoxia-induced cell death. We evaluated the expression and methylation status of BNIP3 gene to better understand the role of epigenetic alteration of its expression in haematopoietic tumours. Methylation of the region around the BNIP3 transcription start site was detected in four acute lymphocytic leukaemia, one multiple myeloma and one Burkitt lymphoma cell lines, and was closely associated with silencing the gene. That expression of BNIP3 was restored by treatment with 5-aza2′-deoxycytidine (5-aza-dC), a methyltransferase inhibitor, which confirmed the gene to be epigenetically inactivated by methylation. Notably, re-expression of BNIP3 using 5-aza2-dC also restored hypoxia-mediated cell death in methylated cell lines. Acetylation of histone H3 in the 5′ region of the gene, which was assessed using chromatin immunoprecipitation assays, correlated directly with gene expression and inversely with DNA methylation. Among primary tumours, methylation of BNIP3 was detected in five of 34 (15%) acute lymphocytic leukaemias, six of 35 (17%) acute myelogenous leukaemias and three of 14 (21%) multiple myelomas. These results suggest that aberrant DNA methylation of the 5′ CpG island and histone deacetylation play key roles in silencing BNIP3 expression in haematopoietic tumours.

As compared to normal tissues, solid tumours are poorly oxygenated and thus contain regions of hypoxia, which provide a physiologically selective pressure for expansion of cells that have acquired antiapoptotic potential (Graeber *et al*, 1996). Haematopoietic tumours growing in bone marrow are also in a hypoxic environment, and several lines of evidence suggest that, like solid tumours, they are dependent on vascular support (Aguayo *et al*, 1999). Indeed, angiogenesis is an important determinant of tumour cell survival under hypoxic conditions, and expression of VEGF is known to be upregulated in acute and chronic myelogenous leukaemia (Aguayo *et al*, 1999; Mayerhofer *et al*, 2002). Little is known, however, about how haematopoietic tumour cells escape apoptosis normally induced by hypoxic stress.

First identified via its interaction with adenovirus E1B 19-kDa and Bcl-2, BNIP3 is one of the Bcl-2 homology 3 (BH3)-only subfamily of Bcl-2 proteins, which display proapoptotic activity (Boyd *et al*, 1994). Forced expression of BNIP3 induces cell death characterised by localisation of the protein at the mitochondria, opening of the permeability transition pore, loss of membrane potential and production of reactive oxygen species (Regula *et al*, 2002; Vande Velde *et al*, 2000). This form of cell death is independent of cytochrome *c* release from mitochondria and caspase activation (Vande Velde *et al*, 2000). Expression of BNIP3, which is dramatically increased in response to hypoxia, is regulated in part by hypoxia-inducible factor-1 (HIF-1), a heterodimeric transcription factor comprised of an oxygen-regulated *α* subunit (HIF-1*α*) and a stale nuclear factor (HIF-1*β*/ARNT), and known to be a mediator of the hypoxic response (Semenza, 1999). Under normoxic conditions, HIF-1*α* is rapidly degraded by proteasome after being targeted for ubiquitination (Maxwell *et al*, 1999). Under hypoxic conditions, degradation of HIF-1*α* is suppressed, and expression of BNIP3 is induced.

There is now compelling evidence that in many human neoplasias, epigenetic alteration plays a key role in silencing genes involved in cell cycle regulation, apoptosis, metastasis and immune responses (Jones and Laird, 1999; Toyota and Issa, 1999; Baylin *et al*, 2001). For example, DNA methylation and histone deacetylation are known to be responsible for silencing various genes in neoplasias (Bird and Wolffe, 1999; Magdinier and Wolffe, 2001; Nguyen *et al*, 2001). However, although changes in DNA methylation are known to play a role in leukaemogenesis (Melki and Clark, 2002), little is known about epigenetic alteration of genes involved in hypoxia. To clarify the molecular mechanism of the resistance to hypoxia-induced apoptosis in haematopoietic tumours, we examined BNIP3 expression in a panel of haematopoietic tumour cell lines and evaluated the DNA methylation and histone acetylation status of the 5′ CpG island of the BNIP3 gene. Our results indicate that aberrant methylation and histone deacetylation of the region around the transcription start site is closely associated with the loss of BNIP3 expression, and that methyltransferase inhibitors restore expression. The use of such drugs may thus represent an effective new approach to the treatment of haematopoietic tumours.

## MATERIALS AND METHODS

### Cell lines and specimens

We used 14 acute lymphocytic leukaemia (ALL) cell lines (BALL1, Jurkat, CCRF-CEM, CCRF-SB, CCRF-HSB2, Molt4, SupT1, PEER, TALL1, Molt3, TOM1, LB804ALL, NALM21 and NAGL1), four multiple myeloma cell lines (KMS12-PE, RPMI8226, HS-Sultan and KHM1B), two Burkitt lymphoma cell lines (Daudi and Raji) and one myeloid leukaemia cell line (K562). All these cells were cultured in appropriate medium. In addition, we analysed 34 primary ALL, 35 primary acute myeloid leukaemia (AML) and 14 primary multiple myeloma specimens, as described previously (Toyota *et al*, 2001; Shen *et al*, 2003).

DNA was extracted from cells and tissue samples using the phenol/chloroform extraction method; total RNA was extracted using ISOGEN (Nippon Gene, Japan) according to the manufacturer’s instructions. To analyse restoration of BNIP3 expression, SupT1, PEER, TALL1, Raji and KHM1B cells were incubated for 72 h with 1 *μ*M 5-aza-2′-deoxycytidine (5-aza-dC) (SIGMA, St Louis, MO, USA). SupT1 cells were treated with 0.2 *μ*M 5-aza-dC, 300 nM trichostatin A (TSA) or both.

### Reverse transcription (RT)–PCR

Total RNA (5 *μ*g) was reverse-transcribed using Superscript III (Invitrogen, Carlsbad, CA, USA) to prepare first-strand cDNA. The primer sequences and PCR parameters used are shown in Table 1. Controls consisted of RNA treated identically but without the addition of reverse transcriptase and are labelled as RT−. The integrity of the cDNA was confirmed by amplifying GAPDH as described previously (Suzuki *et al*, 2000). Samples (10 *μ*l) of amplified product were then subjected to 2.5% agarose gel electrophoresis and stained with ethidium bromide.

Real-time PCR was carried out using an ABI Prism 7000 (Applied Biosystems, Foster City, CA). Accumulation of PCR product was measured in real time as an increase in SYBR green fluorescence and analysed using ABI Prism 7000 SDS Software (Applied Biosystems). Standard curves relating initial template copy number to fluorescence and amplification cycle were generated using the amplified PCR product as a template, and were then used to calculate the mRNA copy number in each sample. The ratios of the intensities of the BNIP3 and GAPDH signals were considered to be a relative measure of the BNIP3 mRNA level in each specimen.

### Western blot analysis

Cells were lysed in ice-cold Tris buffer (20 mM Tris, pH 7.5). containing 137 mM NaCl, 2 mM EDTA, 1% Triton X, 10% glycerol, 50 mM NaF, 1 mM DTT and a protease inhibitor cocktail (Roche Applied Science, Mannheim, Germany). Samples (20 *μ*g) of the cell lysate were then subjected to 10% SDS–PAGE, after which the resolved proteins were transferred to Immobilon-P membranes (Millipore, Bedford, MA). After blocking with 5% nonfat milk and 0.1% Tween-20 in Tris-buffered saline, the membranes were probed with anti-HIF-1*α* (Cell Signaling, Beverly, MA, USA) and anti-BNIP3 mouse monoclonal antibodies (Abcam, Cambridge, UK). The blots were then visualised using enhanced chemiluminescence (Amersham, Bucks, UK).

### Bisulphite treatment

For bisulphite-PCR, genomic DNA was initially treated with sodium bisulphite (SIGMA) as described previously (Clark *et al*, 1994). DNA (2 *μ*g) was denatured for 10 min in 2 M NaOH at 37°C before addition of 30 *μ*l of 10 mM hydroquinone (SIGMA) and 520 *μ*l of 3 M sodium bisulphite (pH 5.0). The mixture was then incubated for 16 h at 50°C. The resultant modified DNA was purified using a Wizard DNA Purification System (Promega, Madison, WI, USA), after which it was again treated with NaOH and precipitated. Finally, the DNA precipitate was resuspended in 20 *μ*l of water and stored at −20°C until used.

### Combined bisulphite restriction analysis

Combined bisulphite restriction analysis (COBRA), a semiquantitative methylation analysis, was carried out as described previously (Xiong and Laird, 1997). PCR was performed in a 50-μl volume containing 1 × PCR buffer (67 mM Tris–HCl, pH 8.8, 16.6 mM (NH_4_)_2_SO_4_, 6.7 mM MgCl_2_ and 10 mM *β*-mercaptoethanol), 0.25 mM dNTP mixture, 0.5 *μ*M each primer and 1.0 U of Hot Start *Taq* polymerase (TaKaRa, Tokyo, Japan). PCR was then carried out using the primer sequences and conditions listed in Table 1. Primers were designed based on the nucleotide sequences obtained from Genbank (AL162274). In total, 20 *μ*l of PCR product were digested with *Afa*I (TaKaRa) and precipitated with ethanol, after which the DNA was subjected to 3% Nusieve gel electrophoresis and stained with ethidium bromide. The intensity of the methylated alleles was calculated by densitometry using a Lane and Spot Analyzer 6.0 (Atto, Tokyo, Japan).

### Bisulphite-sequencing

For bisulphite-sequencing, the PCR products obtained with Bisulphite-PCR were cloned into pCR4.0 vector using a TOPO-TA cloning kit (Invitrogen), after which 10 clones were sequenced for each cell line analysed. The plasmid DNA was then purified using a PI-100 Plasmid Purification System (Kurabo, Tokyo, Japan) and sequenced using a Big Dye Terminator Cycle Sequencing Ready Reaction Kit (Applied Biosystems) with an ABI PRISM 3100 Genetic Analyzer (Applied Biosystems). For direct sequencing, amplified bisulphite-PCR products were electrophoresed in a 1% Seaplaque gel, excised and purified using a PCR Purification System (Promega). The cycle sequencing reaction was carried out as described above.

### Flow cytometry

Jurkat and TALL1 cells were incubated for 96 h under normoxic or hypoxic (1% O_2_, 94% N_2_, 5% CO_2_) conditions, harvested, fixed with 90% ethanol and incubated for 30 min with 2 mg ml^−1^ RNase (SIGMA). In addition, in some instances, TALL1 cells were pretreated for 72 h with 1 *μ*M 5-aza-dC. The cellular DNA was then stained with 50 mg ml^−1^ propidium iodide for 30 min at 4°C, after which numbers of stained cells were determined using fluorescent-activated cell sorting (FACS) on a Becton Dickinson FACScaliber.

### Chromatin immunoprecipitation analysis

Chromatin immunoprecipitation (ChIP) analysis was carried out as described previously (Magdinier and Wolffe, 2001). Briefly, DNA was crosslinked with chromatin by incubating cells in 1.0% formaldehyde for 10 min at 37°C. The cells were then washed with ice-cold PBS containing protease inhibitors and resuspended in lysis buffer (1% SDS, 10 mM EDTA, 50 mM Tris–HCl, pH 8.0 and protease inhibitor). The DNA within the chromatin was then fragmented into 200–1000-bp segments by sonication, after which immunoprecipitation was carried out for 16 h at 4°C with rotation using antiacetylated histone H3 antibody (Upstate Biotechnologies, Lake Placid, NY, USA) as a probe. The resultant immune complexes were collected using protein A-agarose, and the DNA was purified by phenol/chloroform extraction, precipitated with ethanol and resuspended in water. About 1/100 of the precipitated DNA was used for PCR. Real-time PCR was carried out using SYBR Green sequence detection reagents (Applied Biosystems) as shown above. The primers used were ChIP-F and ChIP-R; their sequences and the PCR parameters are shown in Table 1. Each experiment was repeated three times, and the average BNIP3/GAPDH signal ratios are shown on the *Y*-axis.

## RESULTS

**Figure 1:**
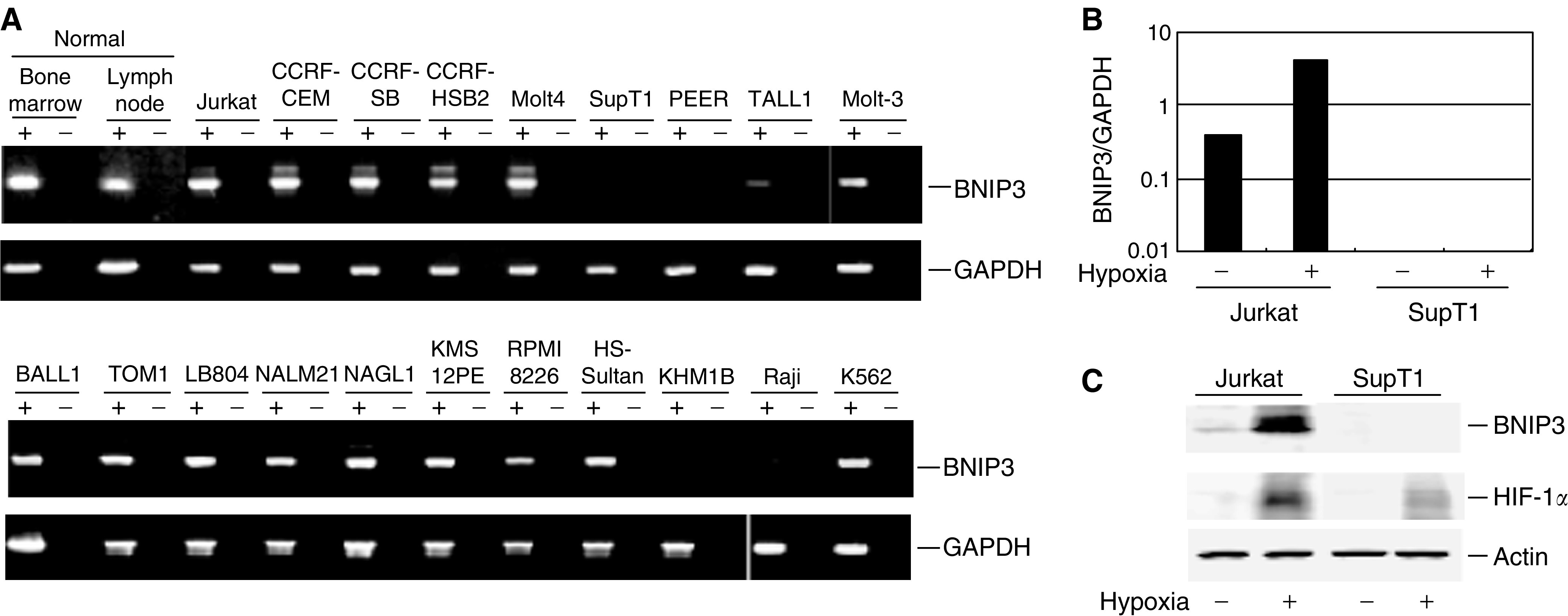
Expression of BNIP3 in haematopoietic tumour cell lines. (**A**) A panel of haematopoietic tumours cell lines was analysed for BNIP3 expression by RT–PCR. Glyceraldehyde-3-phosphate dehydrogenase (GAPDH) served as an internal control for the integrity of the cDNA. Corresponding negative controls (amplification without RT) are shown as RT-negative. Cell lines and tissues used are shown on the top. (**B**) Effect of hypoxia on expression of BNIP3. Real-time PCR was carried out using cDNA from cells subjected to normoxic (20% O_2_, hypoxia−) or hypoxic (1% O_2_, hypoxia+) conditions for 24 h. Signals normalised to GAPDH are shown on the *Y*-axis. Cell lines examined are shown below the columns. (**C**) Western blot analysis of HIF-1*α* and BNIP3. Cells were incubated for 24 h under normoxic (20% O_2_, hypoxia-) or hypoxic (1% O_2_, hypoxia+) conditions, after which the proteins were separated by SDS–PAGE, and HIF-1*α*, BNIP3 and actin proteins were detected using appropriate antibodies.

We initially used RT–PCR to evaluate BNIP3 expression in a panel of 20 haematopoietic tumour cell lines (Figure 1A). Expression of BNIP3 was readily detectable in normal bone marrow and lymph nodes, as well as in 15 of the cell lines tested. Of the remaining five lines, two (TALL1 and Raji) expressed only negligible levels of BNIP3, and three (SupT1, PEER and KHM1B) expressed none at all. The earlier findings that under hypoxic conditions expression of BNIP3 can be induced by the transcription factor HIF-1*α* (Bruick, 2000; Guo *et al*, 2001; Sowter *et al*, 2001) prompted us to evaluate the extent to which expression of BNIP3 in haematopoietic tumour cell lines could be induced by hypoxia. Using real-time PCR with cDNA prepared from Jurkat and SupT1 cells incubated under hypoxic conditions, we found that hypoxia leads to increased expression of BNIP3 in Jurkat cells but not in SupT1 cells (Figure 1B). When we carried out a Western blot analysis to determine whether the absence of BNIP3 expression in SupT1 cells was caused by impaired HIF-1*α* function, we found that hypoxia induced HIF-1*α* expression in both Jurkat and Supt1 cells; thus, the absence of BNIP3 was not caused by a HIF-1*α* deficiency (Figure 1C).

**Figure 2:**
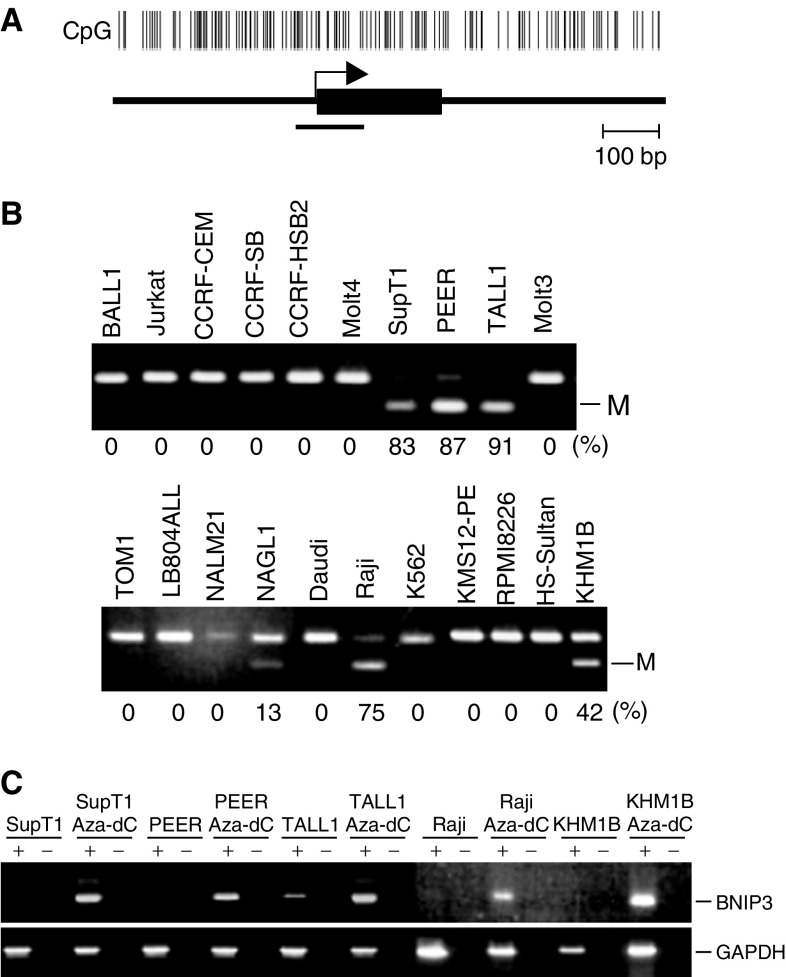
Analysis of BNIP3 methylation in a panel of haematopoietic tumour cell lines. (**A**) CpG island of BNIP3; CpG sites are shown by vertical bars. The region analysed by COBRA is shown by a solid bar. Exon 1 is shown by a solid box on a solid line. The transcription start site is shown by an arrow. (**B**) Aberrant methylation of BNIP3 in haematopoietic tumour cell lines. Methylation of BNIP3 was examined using COBRA. Percentages of methylated alleles are shown below the gels. M: methylated allele. (**C**) Analysis of BNIP3 expression before and after treatment with 5-aza-dC. Cell lines were treated for 96 h with 2.0 *μ*M 5-aza-dC and then harvested, after which RNA was extracted.

Using Blast (http://www.ncbi.nlm.nih.gov/BL AST/) and CpG island Searcher (http://www.uscnorris.com/cpgis lands/), we found that the 5′ region of BNIP3 contains a CpG-rich region that satisfies the criteria for a CpG island (CpG : GpC=0.65, GC%=55%; Figure 2A). Then, to explore the role of BNIP3 methylation in haematopoietic tumours, we first used COBRA, a semiquantitative methylation analysis, to examine the methylation status of the region around the transcription start site in a panel of haematopoietic tumour cell lines. Aberrant methylation of BNIP3 was detected in all five cell lines (SupT1, PEER, TALL1, Raji and KHM1B) that either did not express BNIP3 at all or expressed it only to a negligible degree (Figure 2B). By contrast, methylation of BNIP3 was not detected in cell lines that expressed BNIP3, although NAGL1 showed a low level of methylation but expressed BNIP3, nevertheless. Notably, expression of BNIP3 could be restored in the five methylated cell lines by treating them with the methyltransferase inhibitor 5-aza-dC, which strongly suggests that BNIP3 was epigenetically silenced by methylation in these cells (Figure 2C).

**Figure 3:**
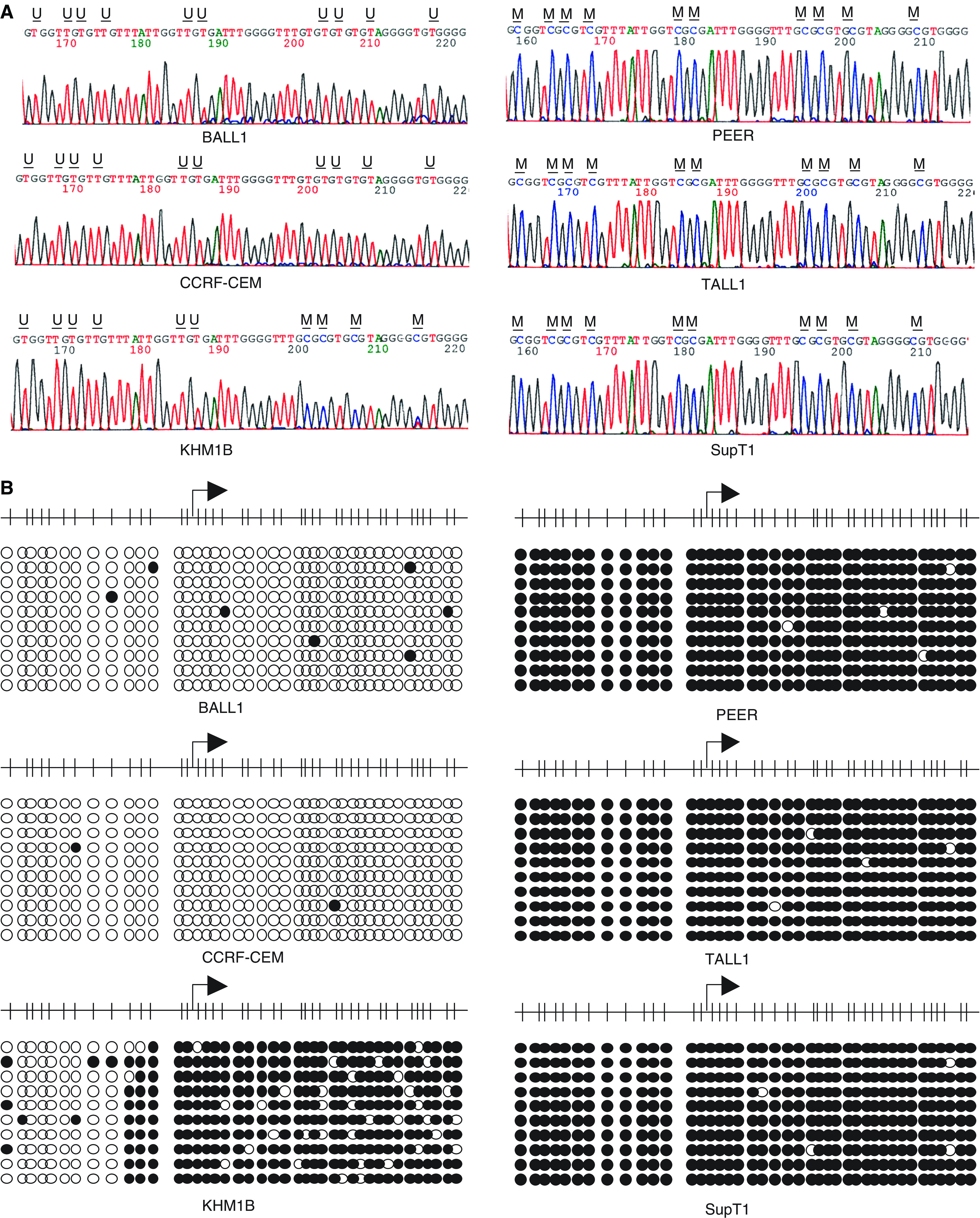
Bisulphite-sequencing of BNIP3. (**A**) Representative results of bisulphite-sequencing. Amplified PCR products were purified from gels and then directly sequenced. The cell lines examined are shown below the column; U, unmethylated CpG sites; M, methylated CpG sites. (**B**) Summary of bisulphite-sequencing. Each circle represents a CpG dinucleotide. Methylation status: open circles, unmethylated; black circles, methylated. At least seven clones were sequenced for each case. The CpG sites in the region analysed are indicated by vertical bar (top).

To examine the methylation status of each CpG dinucleotide within the BNIP3 CpG island, bisulphite-sequencing was carried out in six cell lines (BALL1, CCRF-CEM, KHM1B, SupT1, PEER and TALL1). In BALL1 and CCRF-CEM cells, which COBRA showed to be unmethylated, virtually no methylation was detected within the region analysed (Figure 3A, B). Conversely, in SupT1, PEER and TALL1 cells, which COBRA showed to be highly methylated, virtually all of the CpG sites within the region examined were methylated (Figure 3A, B). KHM1B cells showed a more a heterogeneous methylation pattern (Figure 3A, B).

**Figure 4:**
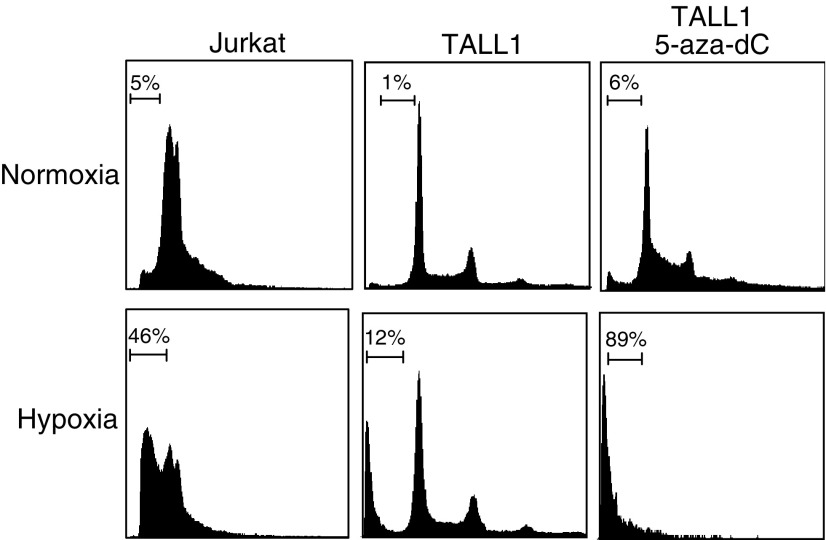
Restoration of hypoxia-mediated apoptosis by treatment with 5-aza-dC. TALL1 cells were treated with either mock or 1 *μ*M 5-aza-dC for 72 h followed by incubation under normoxic or hypoxic conditions for 96 h. Percentages of apoptotic cells were determined by flow cytometry. The unmethylated Jurkat cell line served as a control.

To determine the extent to which the silencing of BNIP3 affected hypoxia-mediated apoptosis, we examined the effect of 5-aza-dC on the incidence hypoxia-mediated cell death among TALL1 cells, which do not otherwise express BNIP3 as a result of the gene's methylation (Figure 4). We found that hypoxia readily induced cell death among control Jurkat cells, which do express BNIP3. Among TALL1 cells, pretreatment with 5-aza-dC had no significant effect on the incidence of cell death under normoxic conditions. Under hypoxic conditions, by contrast, significant numbers of apoptotic cells were detected following pretreatment with 5-aza-dC, which is indicative of the role played by epigenetic silencing of BNIP3 in protecting cells from hypoxia-mediated apoptosis.

**Figure 5:**
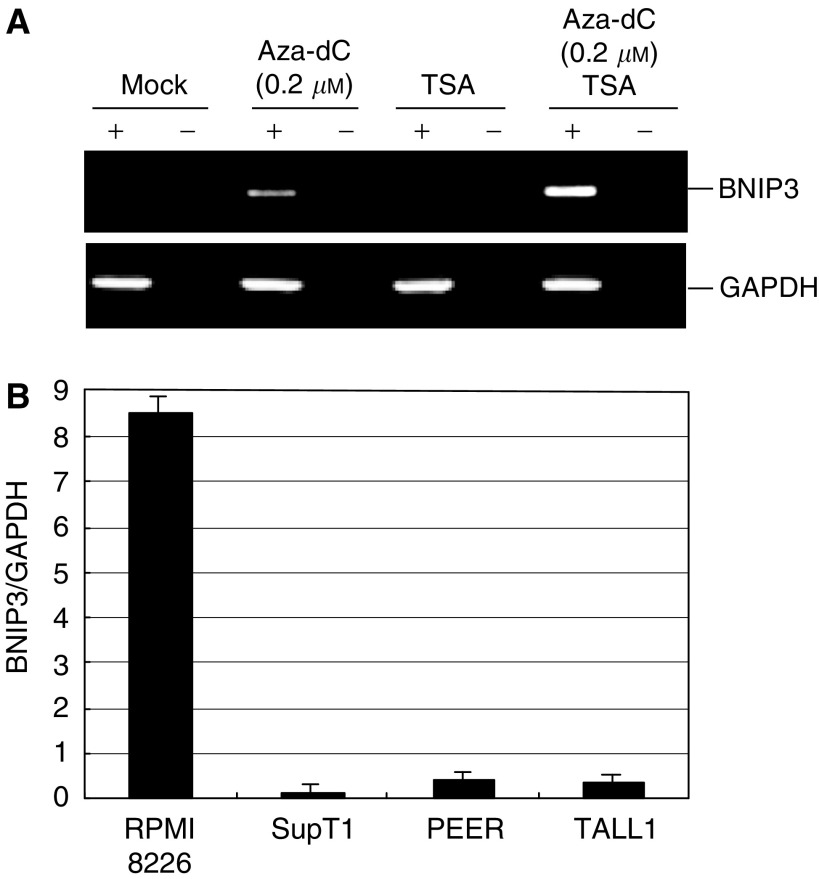
The role of histone deacetylation in silencing BNIP3 gene expression. (**A**) Effects of DNA methyltransferase and/or histone deacetylase inhibitor on the expression of BNIP3. SupT1 cells, which show BNIP3 methylation, were treated with 0.2 *μ*M 5-aza-dC, 300 nM TSA or both. (**B**) Histone acetylation examined by ChIP followed by PCR. Chromatin immunoprecipitation analysis was carried out using DNA precipitated with antiacetylated histone H3 antibody; the bars show the levels of histone acetylation determined by real-time-PCR normalised to the GAPDH signal. The cell lines examined are indicated below.

Methylation-dependent gene silencing is reportedly associated with altered chromatin structure involving deacetylation of histone (Jones and Baylin, 2002). Therefore, to assess the role of histone deacetylation in the silencing of BNIP3, we treated the methylated SupT1 cell line with 5-aza-dC and/or TSA, a histone deacetylase inhibitor (Figure 5A). Treating the cells with a low dose (0.2 *μ*M) of 5-aza-dC induced only a small amount of BNIP3 expression. However, addition of TSA (300 nM) to the 5-aza-dC had a synergistic effect that markedly increased gene expression, indicating a role for histone deacetylation in silencing of BNIP3 gene expression. We then examined the acetylation status of the BNIP3 CpG island in more detail using ChIP assays with anti-histone H3 antibody and found that acetylation of histone H3 in the region around the transcription start site correlated directly with gene expression and inversely with DNA methylation (Figure 5B).

**Figure 6:**
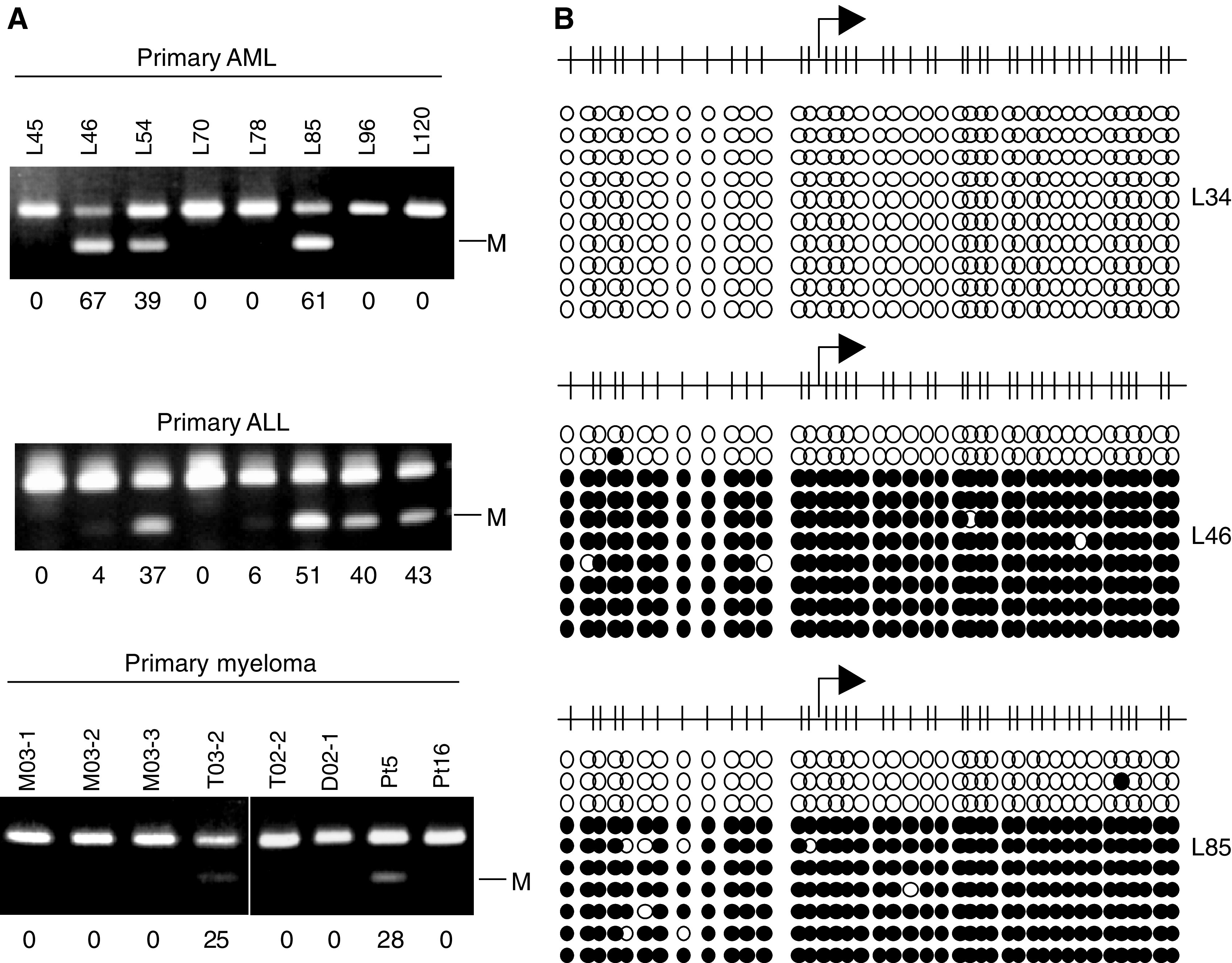
Analysis of BNIP3 methylation in a panel of primary haematopoietic tumours. (**A**) Methylation of BNIP3 examined using COBRA. Tumour type is shown above the gels. Percentages of methylated alleles were calculated by densitometry and are shown below the gels. (**B**) Summary of bisulphite-sequencing. Each circle represents a CpG dinucleotide. Methylation status: open circles, unmethylated; black circles, methylated. The cases examined are shown on the right.

Finally, we determined the extent to which BNIP3 is methylated in primary tumours by examining the methylation status of BNIP3 in a panel of primary leukaemia specimens. Using COBRA, methylation of BNIP3 was detected in five of 34 (15%) ALL specimens, in six of 35 (17%) AML specimens and in three of 14 (21%) multiple myeloma specimens (Figure 6A). These findings were then confirmed by bisulphite-sequencing of the amplified PCR products. All of the cases found to be methylated using COBRA were found to be methylated at all of the CpG sites examined (Figure 6B, C).

## DISCUSSION

Tumour cells have an ability to escape apoptosis induced by stresses such as DNA damage, growth factor withdrawal and hypoxia. Resistance to hypoxia-induced apoptosis, in particular, is an important factor in the progression of human neoplasias and in the development of resistance to chemotherapy (Harris, 2002). This is because selective elimination of apoptosis-sensitive cells leads to expansion of cells that are more resistant to treatment and thus contribute to tumour relapse (Schmaltz *et al*, 1998). In contrast to solid tumours, little is known about how the cells of haematopoietic tumours escape apoptosis induced by hypoxic stress. In the current study, however, we have shown for the first time that aberrant methylation of BNIP3 and histone deacetylation are involved in silencing the gene in a subset of ALLs, AMLs and multiple myelomas. This suggests that tumour cells that do not express BNIP3 have a growth advantage related to their ability to escape apoptosis induced by hypoxia.

BNIP3 was originally identified using a yeast two-hybrid screen for proteins that bind to the adenoviral E1B 19-kDa protein (Boyd *et al*, 1994). The protein contains a BH3 domain that induces apoptosis (Yasuda *et al*, 1998) and a C-terminal transmembrane domain that is required for both its mitochondrial localisation and its proapoptotic activity (Chen *et al*, 1997, 1999; Yasuda *et al*, 1998); it has been suggested that BNIP3 mediates a necrosis-like cell death by causing mitochondrial dysfunction (Harris, 2002). Hypoxia induces expression of a group of genes, among which is the transcriptional regulator HIF-1. BNIP3 is a principle target of HIF-1, and the region containing BNIP3's transcription start site also contains HIF-1-binding sequences (Bruick, 2000; Sowter *et al*, 2001). It is noteworthy that because HIF-1 is involved in both survival and apoptosis of tumour cells, abrogation of the apoptotic pathway caused by silencing BNIP3 may enhance HIF-1-induced survival signals within tumours.

Expression of BNIP3, which is induced by hypoxic stimuli (Guo *et al*, 2001), has been detected in several human cancer cell lines and cardiac myocytes subjected to hypoxia (Crow, 2002; Kubasiak *et al*, 2002; Regula *et al*, 2002). In addition, Sowter *et al* (2003) recently reported that BNIP3 is highly expressed in the hypoxic regions of high-grade breast cancer. Future clinicopathological analysis may clarify whether there is a specific phenotype for tumours with BNIP3 methylation.

In the present study, we have shown that the silencing of BNIP3 is the result of DNA methylation of its 5′ CpG island and histone deacetylation. The fact that in methylated cell lines BNIP3 expression was restored by 5-aza-dC, a methyltransferase inhibitor, confirms that inactivation of the gene was caused by DNA methylation and not by, for example, the loss of transcription factors. DNMT1 and DNMT3b are known to catalyse DNA methylation in cancer cells (Rhee *et al*, 2002; Toyota *et al*, 2003); the mechanism by which DNA methylation represses gene expression is not fully understood, however, although histone deacetylation reportedly plays a key role (Bird and Wolffe, 1999; Cameron *et al*, 1999; Nguyen *et al*, 2001). Consistent with that finding, our ChIP assays showed histone H3 to be deacetylated in cell lines where BNIP3 is silent. It may be that dense methylation of the BNIP3 CpG island attracts methyl-CpG-binding proteins, such as MeCP2 and MBD2, which in turn recruit histone deacetylases (Jones and Baylin, 2002). Recent reports also suggest that histone methylation may be involved in DNA methylation-dependent gene silencing (Fahrner *et al*, 2002; Nguyen *et al*, 2002; Kondo and Issa, 2003). Further study will be necessary to clarify the precise mechanism by which CpG methylation silences BNIP3.

In summary, we have shown that DNA methylation and histone deacetylation play key roles in silencing BNIP3 gene expression in haematopoietic tumours. Inhibition of DNA methylation and histone deacetylation act synergistically to induce gene expression, suggesting that BNIP3 may be an effective molecular target for treating a subset of haematopoietic tumours through activation of apoptosis by methyltransferases and histone deacetylase inhibitors.

## Figures and Tables

**Table 1:** Primer sequences for BNIP3 analysis

		**Annealing °C (cycles)**	**Size (bp)**
COBRA	F: 5′-TTYGGTYGGAGGAATTTATAGGGTAG-3′	58(3), 56(4), 54(5), 52(26)	156
	R: 5′-CCCTCRCCCACCRCAAAAC-3′		
Bisulphite-sequencing	F: 5′-GATATGGYGTTAGAGGGTAATTG-3′	58(3), 56(4), 54(5), 52(26)	294
	R: 5′-CCCTCRCCCACCRCAAAAC-3′		
RT–PCR	F: 5′-CCACCTCGCTCGCAGACACCAC-3′	66(3), 64(4), 62(5), 60(23)	317
	R: 5′-GAGAGCAGCAGAGATGGAAGGAAAAC-3′		
Real-time PCR	F: 5′-GGACAGAGTAGTTCCAGAGGCAGTTC-3′	60 (40)	90
	R: 5′-GGTGTGCATTTCCACATCAAACAT-3′		
ChIP			
BNIP3	F: 5′-CCGCGCCGCCTCCTCCGCCTCAC-3′	70 (35)	159
	R: 5′-GCTCCGACCTCCGCTTTCCCACCGCC-3′		
GAPDH	F: 5′-TCGGTGCGTGCCCAGTTGAACC-3′	62 (35)	246
	R: 5′-ATGCGGCTGACTGTCGAACAGGAG-3′		

Y=C or T, R=A or G; ChIP=Chromatin immunoprecipitation; GAPHD=glyceraldehydes-3 phosphate dehydrogenase; COBRA=combined bisulphite restriction analysis.
